# The effect of hand motion and object orientation on the automatic detection of orientation: A visual mismatch negativity study

**DOI:** 10.1371/journal.pone.0229223

**Published:** 2020-02-26

**Authors:** Bela Petro, Petia Kojouharova, Zsófia Anna Gaál, Boglárka Nagy, Petra Csizmadia, István Czigler

**Affiliations:** 1 Doctoral School of Psychology, Eötvös Loránd University, Budapest, Hungary; 2 Institute of Psychology, ELTE Eötvös Loránd University, Budapest, Hungary; 3 Research Centre for Natural Sciences, Institute of Cognitive Neuroscience and Psychology, Budapest, Hungary; 4 Department of Cognitive Science, Faculty of Natural Sciences, Budapest University of Technology and Economics, Budapest, Hungary; Universidad de Salamanca, SPAIN

## Abstract

We investigated the effects of voluntary hand movements and continuously present objects on the automatic detection of deviant stimuli in a passive oddball paradigm. The visual mismatch negativity (vMMN) component of event-related potentials (ERPs) was measured as the index of automatic deviant detection. The stimuli were textures consisting of parallel, oblique bars with frequent (standard) and infrequent (deviant) orientation. Traditional vMMN was measured by the difference between ERPs to frequent (standard) and infrequent (deviant) textures. Additionally, we measured ‘genuine’ vMMN by comparing the ERPs to deviant and control textures in the equal probability procedure. Compatible and incompatible hand movement directions to the standard texture had no influence on ‘traditional’ vMMN and elicited no ‘genuine’ vMMN. However, the deviant texture elicited ‘genuine’ vMMN if the orientation of a continuously present rectangle was different from the standard (and identical to the deviant) texture orientation. Our results suggest that the direction of voluntary hand movement and the orientation of task-irrelevant visual patterns do not acquire common memory representation, but a continuously present object contributes to the detection of sequential regularity violation.

## Introduction

Visual mismatch negativity (vMMN), a component of event-related potentials (ERPs), is elicited by stimuli violating the regularity of sequential stimulation. The regular sequence can be defined by particular values of visual features, like orientation, spatial frequency, color, etc., perceptual categories (e.g., symmetry, numerosity, object-related regularities), higher-order visual (e.g., facial emotion, gender, left vs. right hand) and sequential characteristics, and even semantic characteristics. VMMN is generated in visual brain areas (within the occipital, temporal or parietal cortices). According to MEG studies, within the occipital cortex the middle occipital gyrus is involved [1,2]. Some research suggests that anterior structures are also involved (e.g., [3]). Importantly, according to an MEG study [4] the occipital source of the vMMN was different from the source of exogenous posterior activity. VMMN is elicited by task-irrelevant stimuli. Accordingly, this ERP component is considered as an index of automatic change detection. For reviews on vMMN see [5–7].

The first aim of the present research was to investigate the possibility of vMMN modulation by motor activity as suggested by theories of common coding of perception and action. The theory claims that seeing an event activates the action associated with that event, and performing an action activates the associated perceptual event (e.g., [8,9]). The second aim was to explore the possibility of vMMN modulation by the presence of an object in the visual field. This is because the features of an object (e.g., orientation) may adapt the visual structures similar to those involved in processing of the vMMN related stimuli, therefore change the sensibility of these structures for particular visual features (stimulus-specific adaptation) [10].

Effects of voluntary motion on ERP activity is investigated in various topics. Some studies examined the effects of motor activity on the sensory components of ERPs. As a typical finding in the auditory modality, movement-contingent sounds that follow voluntary movements elicit the N1 component with reduced amplitude, i.e., a correlate of perceptual suppression (e.g., [11,12]). The investigation of this suppression effect is based on the ‘forward model’: The results of voluntary movements are anticipated, and the match between anticipated and incoming stimuli results in a reduced ERP amplitude. However, this explanation of the effect is equivocal [13]. Furthermore, in the visual modality the results are rather ambiguous (see [14] for a review of the literature). Another topic of action-perception interaction is the investigation of the effect of action intention on the allocation of visual attention. The findings of this line of research indicate that the goal of action may facilitate attention capture of visual features connected to the type of motion [15]. Wykowska and Schubö [15] obtained the modulation of both early (P1) and later (N2pc) components, when the action cue preceded a search target that corresponded to the cues (grasping cue and size-related target and pointing cue with location target). According to this result both the sensory (reflected by the P1) and the attentive search-related (reflected by the N2pc) ERP components were sensitive to the action-perception contingency, i.e., motion cues influenced the intentional weighting mechanisms [16].

In vMMN studies the possible relationship between voluntary movements and the vMMN-related stimuli is different from that in attentional studies. This is because the vMMN-related stimuli cannot be attributed to the consequence of movements, as they are not related to the movements. However, in case of an interaction between the representation of movement attributes and the actual visual stimulation (independent of motion), one may expect that repeated unidirectional movements lead to the adaptation of the orientation-specific visual structures. In this way, a movement direction identical to the frequent (standard) stimuli would make the opposite (deviant) orientation more salient. In fact, in a previous study we obtained larger vMMN when the vMMN-related stimuli were in the color domain and the task required orientation discrimination than in case of color-related vMMN together with color discrimination task; and also in the reverse case, we observed larger vMMN to orientation deviancy in a task demanding color discrimination than in a task demanding orientation discrimination [17]. Accordingly, we expected larger vMMN when movement direction and standard orientation match than in case of a movement direction and standard orientation mismatch. In Experiment 2 the required movement direction was cued by a continuously presented rectangle. This way we investigated the possible effect of orientation-specific adaptation on vMMN. We have to emphasize that in the present study we investigated the effects of concomitant hand movements and not the effects of initiating voluntary hand movements on vMMN.

## Experiment 1

In our experiments the participants moved a small disc on a display back and forth with a computer mouse between two targets. In Experiment 1 the required motion was cued by two circles as the endpoints of movements. The vMMN-related stimuli were background textures consisting of parallel, oblique bars with frequent (standard) and infrequent (deviant) orientation. We expected the emergence of a Deviant *minus* Standard difference (‘traditional’ vMMN) in the 100–350 ms latency range over the posterior locations [17–21]. To separate the ERP changes of the standard (stimulus specific adaptation) from the deviant-related changes (‘genuine’ vMMN), we applied the equal probability control procedure [20,22]. The Deviant *minus* Standard and Deviant *minus* Control differences were expected over the posterior EEG locations. Accordingly we created an occipital ROI (O1, Oz, O2 locations).

### Methods

#### Participants

Twenty-four right-handed students (19 female, 5 male; mean age = 22.4 years, *SD* = 2.1) with normal or corrected-to-normal vision participated in the experiment for course credit. Written informed consent was obtained from all participants prior to the experimental procedure. The study was conducted in accordance with the Declaration of Helsinki and approved by the United Ethical Review Committee for Research in Psychology (EPKEB).

#### Stimuli

The stimuli were displayed on a 24-in. LCD monitor (Asus VS229na) with a 60 Hz refresh rate. All stimuli and task-related objects appeared in white (257 cd/m^2^) on a light grey background (42 cd/m^2^). The stimuli were generated and presented by a script written in Matlab (version 2015a, MathWorks, Natick, MA). One centimeter of hand movement (mouse movement) corresponded to 3 centimeters of disc movement on the display. The diameter of the moving disc was 0.11° (from a 144-cm viewing distance). The diameter of the two target circles was 0.77° and the distance between their centers was 5.5°. The movement directions were either in 26° (from bottom-left to top-right and back) or in 170° (from bottom-right to top-left and back) in different blocks. According to informal practice these orientations were comfortable for moving the mouse. A white fixation point was presented in the middle of the screen (diameter: 0.05°), equidistant from the two target circles. The ERP-related stimuli consisted of 95–100 white, parallel bars appearing simultaneously at random locations on the screen excluding the two target circles and the area between them. The length of the individual bars was 1°, and their width was 0.05°. Together the bars made up textures. In the Oddball blocks the texture orientations were identical to or different from the movement directions (see Procedure for details). In the Control blocks the bar textures appeared at equal probability and in random order in the following orientations: 26, 46.6, 67.1, 87.7, 108.3, 128.9, 149.4, 170 degrees.

#### Procedure

**Fig 1:**
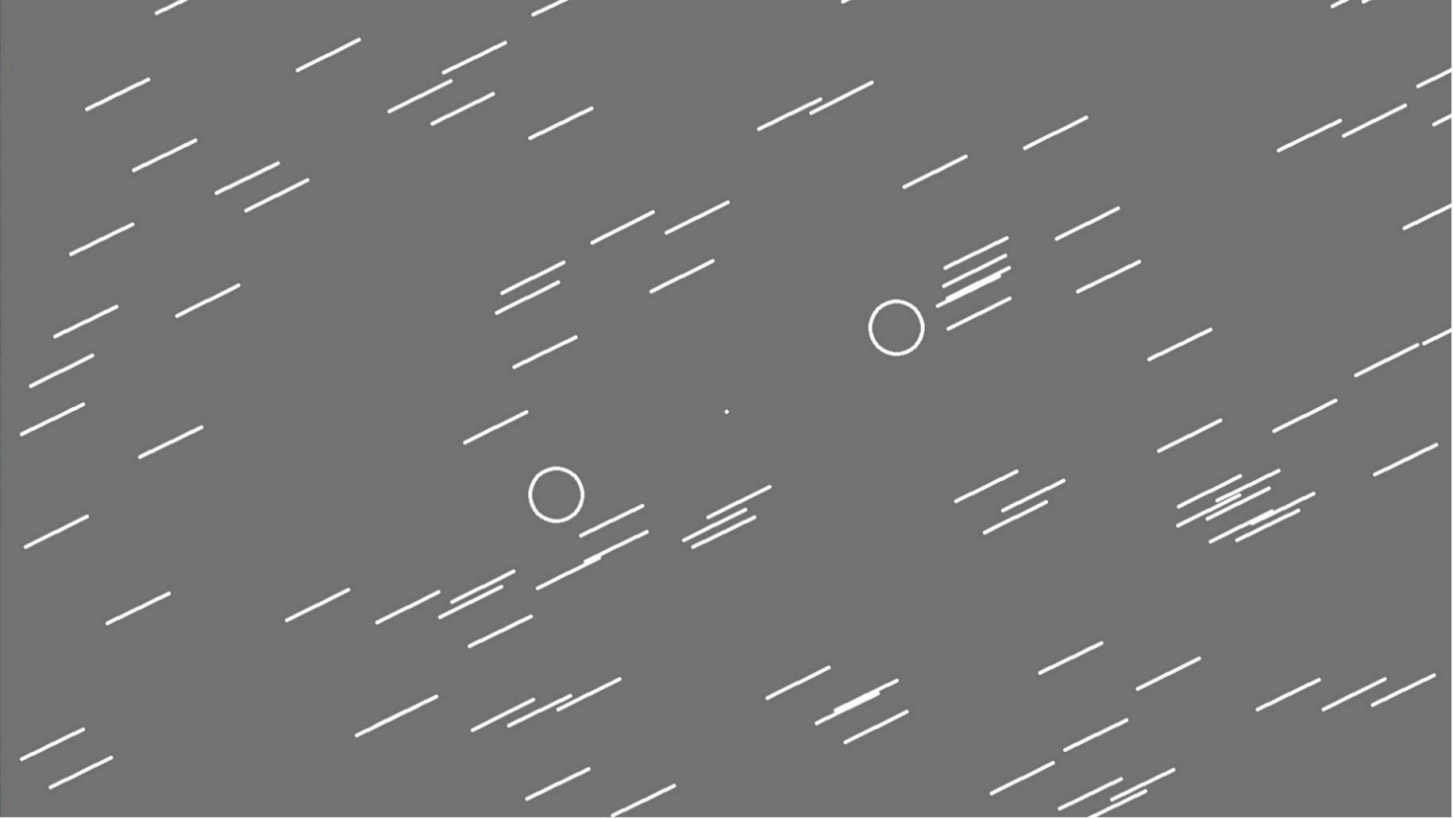
An example of the stimulus display from Experiment 1. Mouse movement 26° - Standard texture 26°.

Participants were seated in a dark, electrically shielded and sound-attenuated room. They were instructed to keep their eyes on the fixation point during the experimental blocks and to move the disc with the mouse back and forth between the target circles as fast and accurately as possible. The experiment started with a one-minute practice block to ensure that the participant fully understood the task. The stimulus duration of the bar texture was 100 ms, the inter-stimulus intervals were 475 ms (with +/-25 ms jitter in 16.6 ms steps). In a particular movement direction–texture orientation oddball combination there were 700 standard and 100 deviant stimuli (87.5 vs. 12.5%), and the presentation order was random. We divided these combinations into two blocks of four minutes to avoid fatigue caused by the mouse movement. These blocks were presented one after the other. After each block feedback was given indicating how many times the participant managed to move the disc between the target circles. Within a session there were four oddball combinations: 1. Mouse movement 26° - Standard texture 26°; 2. Mouse movement 170° - Standard texture 170°; 3. Mouse movement 26° - Standard texture 170°; 4. Mouse movement 170° - Standard texture 26°. The orientation of the deviant texture was 26° in case of a 170° standard texture, and *vice versa*. Furthermore, there were two Control blocks: 1. Control with Mouse movement 26°; 2. Control with Mouse movement 170°. The presentation order of the four Oddball and two Control combinations was counterbalanced within the sample. Oddball combinations 1 and 2 constituted the ‘Same condition’, combinations 3 and 4 the ‘Opposite condition’, and the two control combinations made up the ‘Control condition’. Fig 1 shows an example of the stimulus display.

#### Measurement of electrical brain activity

Electrical brain activity was recorded from 32 locations according to the extended 10–20 system (BrainVision Recorder 1.21.0303, ActiChamp amplifier, Ag/AgCl active electrodes, EasyCap (Brain Products GmbH), sampling rate: 1000 Hz, DC-70 Hz online filtering). The reference electrode was on the nose tip, and the ground electrode was placed on the forehead (AFz). Both horizontal and vertical electrooculogram (HEOG and VEOG) were recorded with bipolar configurations between two electrodes (placed lateral to the outer canthi of the two eyes and above and below the left eye, respectively). The EEG signal was bandpass filtered offline with a non-causal Kaiser-windowed Finite Impulse Response filter (low pass filter parameters: 30 Hz of cutoff frequency, beta of 12.265, a transition bandwidth of 10 Hz; high pass filter parameters: 0.1 Hz of cut off frequency). Stimulus onset was measured by a photodiode, providing exact zero value for averaging. Epochs ranging from –100 to 450 ms relative to the onset of stimuli were extracted for further analysis, separately for standards, deviants and control. The first 100 ms of each epoch served as the baseline. Epochs with larger than 100 μV voltage change at any electrode were considered artefacts and rejected from further processing.

To measure deviant-related activities we calculated Deviant *minus* Standard and Deviant *minus* Control difference potentials separately for the Same and for the Opposite conditions. Mean activities of both differences in the occipital ROI were measured with one-sample t-tests in comparison to zero, and compared in ANOVAs with within-subjects factors of Difference (Deviant *minus* Standard, Deviant *minus* Control) and Condition (Same, Opposite) in the 100–150, 150–200 and 200–350 ms time windows. The choice of these time windows is based on a priori observations. The first time window corresponds to the latency range of the early Deviant *minus* Standard difference. In a related study [23] after the onset of texture patterns vMMN emerged within the 100–150 ms range. The second one corresponds to the range of the posterior N1 component [10,18–21,23–25], whereas in the latest range, a Deviant *minus* Control difference was recorded by Kimura et al. [20]. We chose this calculation to reduce the number of comparisons [26]. For post-hoc comparisons we applied the Tukey HSD test. Effect size was calculated as η_p_^2^. We used the Statistica package (Version 13.4.0.14, TIBCO Software Inc.) for statistical analysis.

Behavioral performance was measured as the number of correct movements within the blocks. The task script was written in such a way that the number of correct moves increased only if both target areas were entered one after the other. We calculated a two-way ANOVA with factors of Condition (Same, Opposite, Control), and Block (First and Second block of the same condition).

### Results

#### Behavioral performance

Table 1 shows the average number of movements in the first and second blocks of the session for movements corresponding to the standard (Same), deviant (Opposite) and variable (Control) orientation of the texture elements. According to a two-way ANOVA with factors of Condition (Same, Opposite, Control) and Blocks, both main effects were significant, F(2,46) = 3.97, ε = 0.90, η_p_^2^ = 0.15, p = 0.030 and F(1,23) = 9.33, η_p_^2^ = 0.29, p = 0.006. Performance increased in the second block, and it was larger in the Control condition. Participants completed 2.01 correct moves/second on average during the whole session.

**Table 1:** Behavioral performance in Experiment 1.

	Same	Opposite	Control
first	444.0 (24.1)	443.1 (26.4)	475.8 (26.7)
second	466.5 (27.9)	461.8 (25.4)	491.5 (27.8)

Mean number of movements in the Same, Opposite and Control conditions, in the first and second blocks (S.E.M. in parenthesis).

#### Event-related potentials

**Fig 2:**
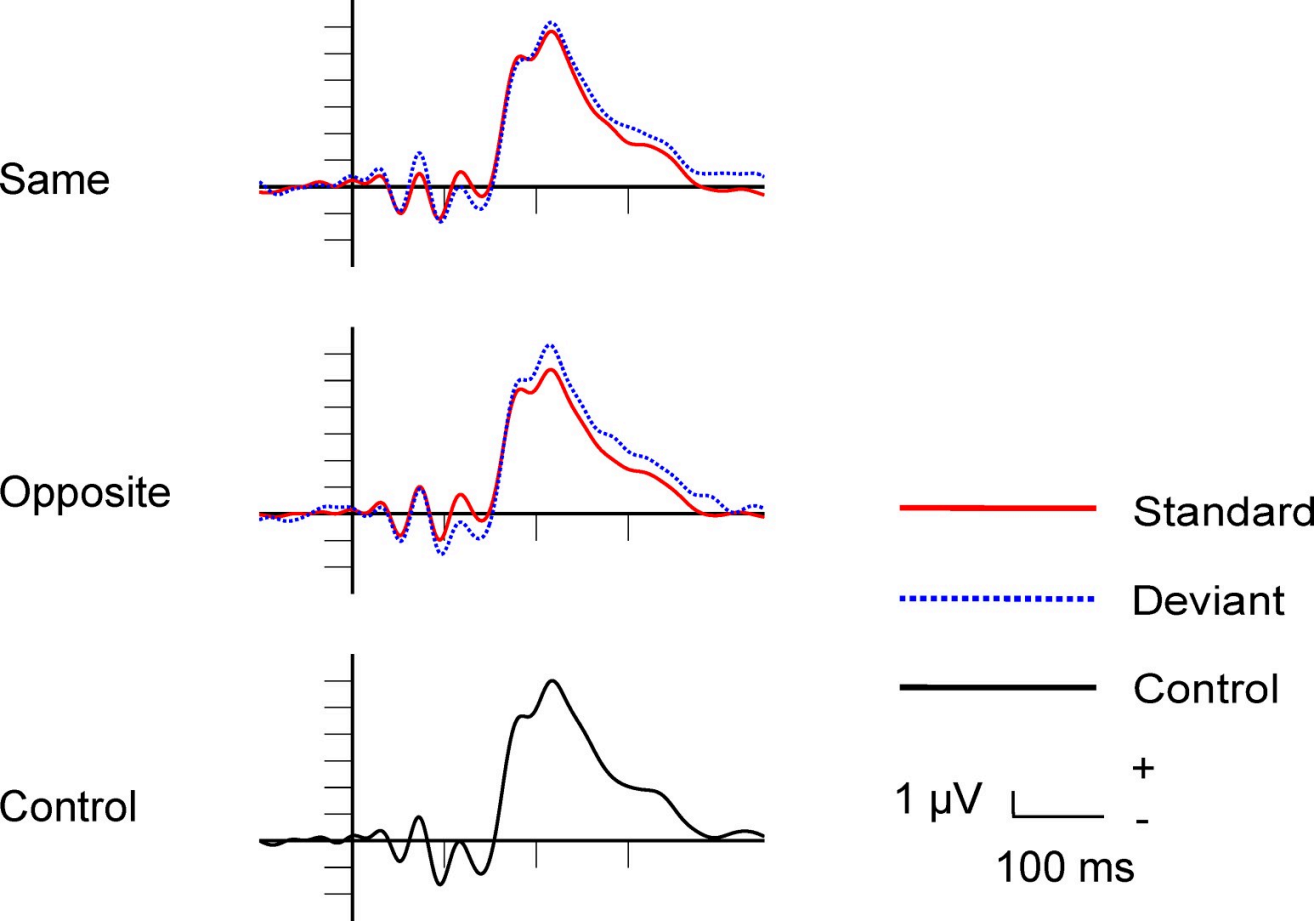
Standard, deviant and control grand-average ERPs in the Same, Opposite and Control conditions in Experiment 1.

The average rejection rate of epochs (eye-blink, eye movement) was 13.19%. Fig 2 shows the standard, deviant and control ERPs in the occipital ROI in the Same, Opposite and Control conditions. The characteristic exogenous components, early positivity (P1/C1), followed by a double negativity (C2/N1) and a positive peak (P2) are present in the ERPs.

#### Difference potentials

**Fig 3:**
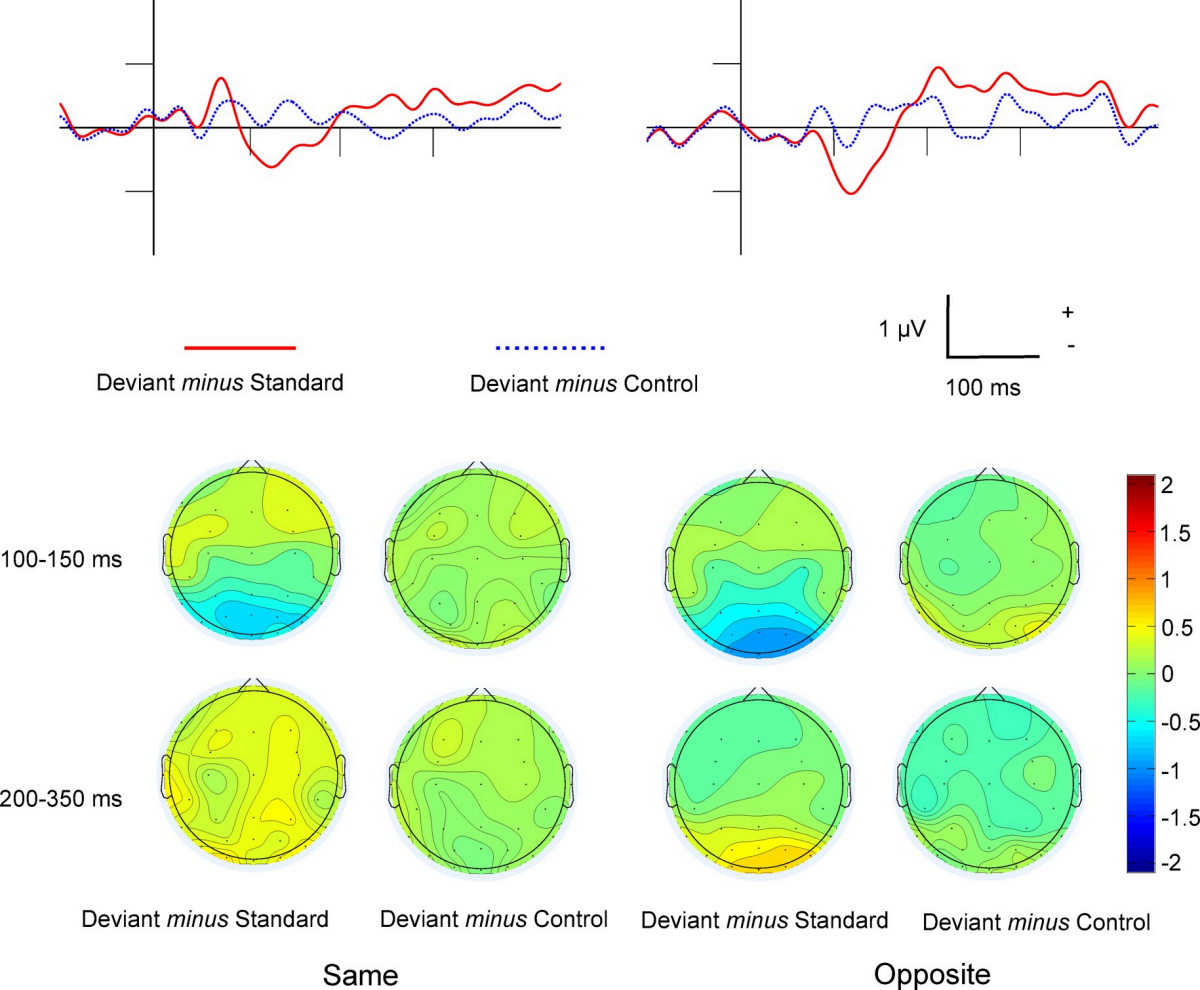
Experiment 1: Difference potentials and surface distributions. The Deviant *minus* Standard and Deviant *minus* Control difference potentials in the occipital ROI and the surface distributions in the Same and Opposite conditions.

Fig 3 shows the Deviant *minus* Standard and Deviant *minus* Control difference potentials in the occipital ROI as well as the surface distributions. Table 2 shows the mean amplitude values of the 100–150, 150–200 and 200–350 ms ranges, and the significance levels of one-sample t-tests in comparison to zero. In the Deviant *minus* Standard difference a negative peak emerged within the 100–150 ms range. The appearance of such negative difference potentials was an expected result of the study. Furthermore, in the 200–350 ms range the difference was positive. However, as Table 2 shows, in the Deviant *minus* Control differences we obtained neither negativity nor positivity.

**Table 2:** Amplitude values of the difference potentials in Experiment 1.

	Deviant *minus* Standard		Deviant *minus* Control	
	Same	Opposite	Same	Opposite
100–150 ms	-0.49 (0.22)*	-0.83 (0.23)**	0.22 (0.22)	0.01 (0.17)
150–200 ms	-0.16 (0.22)	0.16 (0.27)	0.18 (0.20)	0.34 (0.25)
200–350 ms	0.40 (0.19)*	0.67 (0.23)**	0.05 (0.18)	0.15 (0.21)

Mean amplitude values (μV) of the difference potentials in the 100–150, 150–200 and 200–350 ms range (*S*.*E*.*M*. in parenthesis) in the occipital ROI.

*p<0.05 in t-tests, in comparison to zero.

**p<0.01 in t-tests, in comparison to zero.

According to the two-way ANOVA with factors of Difference and Condition, in the 100–150 ms range only the Difference main effect was significant, F(1,23) = 31.516, ƞ_p_^2^ = 0.58, p<0.0001. The difference potential amplitude in the Deviant *minus* Standard difference was -0.66 μV, whereas in the Deviant *minus* Control difference it was 0.11 μV. In the 150–200 ms epoch we obtained no significant difference. In the 200–350 ms range the main effect of Difference was significant again, F(1,23) = 15.490, ƞ_p_^2^ = 0.40, p<0.001. The Deviant *minus Standard* difference was more positive (0.54 μV) than the Deviant *minus* Control difference (0.10 μV).

### Discussion

In this experiment participants continuously moved a disc with the mouse in a direction corresponding to the orientation of frequent (standard) task-irrelevant bars, or the movement direction differed from the standard orientation of bars. This manipulation had no effect on the emergence of a negative Deviant *minus* Control difference potential in the 100–150 ms range. Although the expected negative difference potential emerged (regardless of condition), the equal probability procedure eliminated the negativity, showing that this negativity cannot be considered as ‘genuine’ vMMN. In this respect the results are similar to those reported by Kimura et al. [20] and File et al. [19]. The lack of difference between Deviant and Control conditions, together with the negativity in the Deviant *minus* Standard difference is attributed to the adaptation of processes underlying the standard-related ERPs (e.g., [27]). In the auditory modality this explanation fits the ERP recordings, because the putative adaptation effect corresponds to the latency range of the N1 component [22]. However, Ruhnau et al. [28] recorded N1 in a considerably earlier range than the latency of the negative difference potentials (MMN) using both the equal probability and cascadic control procedures. (The cascadic control uses equal probabilities, but the various stimuli are presented in regular sequence. This control method was introduced into the visual MMN research in a study by File et al. [19].) In the visual modality Kimura et al. [20] obtained similar N1 latency to the latency of the earlier Deviant *minus* Standard negativity. They explained it as an adaptation of the posterior N1. However, in other studies with orientation deviancy the latency range of difference potentials did not correspond to any well-defined ERP components [18]. As for the present study, the Deviant *minus* Standard difference potential covered a latency of both the earlier and the later negativity, i.e., the difference did not correspond to any particular ERP component.

The negative difference potential was followed by a posterior positivity. The emergence of a positivity in vMMN studies is not unprecedented [29,30]. The equal probability control procedure eliminated this positivity, indicating that it was an adaptation-like phenomenon.

Behavioral results indicated a slight practice effect, and a facilitative effect of variable task-irrelevant stimuli. The reliability of this performance variability was checked in Experiment 2.

## Experiment 2

In Experiment 2 we added a continuously presented outline of an oblique rectangle to the display. The rectangle cued the movement direction demand. This way we attempted to facilitate the adaptation of orientation-specific structures, therefore we expected larger contrast between the standard and the deviant, and as a consequence, the possibility of recording ‘genuine’ vMMN. Accordingly, we hypothesized the emergence of vMMN in the Same condition (i.e., identical rectangle orientation and the orientation of the oddball standard).

### Methods

#### Participants

Twenty-six right-handed students (13 female, 13 male; mean age = 22 years, *SD* = 2.5) with normal or corrected-to-normal vision participated in the second experiment for course credit. Written informed consent was obtained from all participants prior to the experimental procedure. The study was conducted in accordance with the Declaration of Helsinki and approved by the United Ethical Review Committee for Research in Psychology (EPKEB).

#### Stimuli and procedure

**Fig 4:**
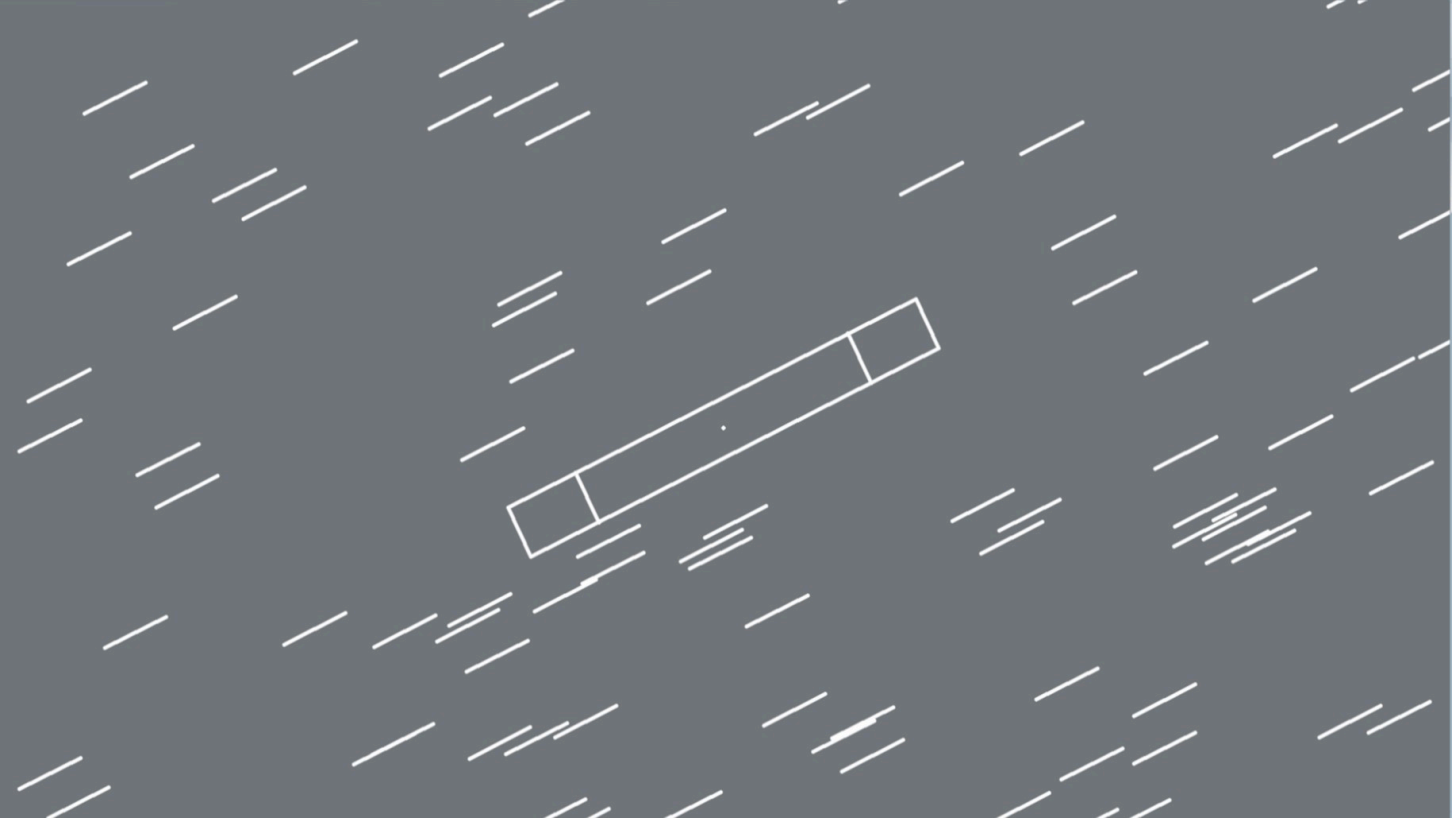
An example of the stimulus display from Experiment 2. Mouse movement 170° - Standard texture 26°.

The only difference in the stimulus display between Experiment 1 and 2 was the replacement of the two target circles with the outline of a rectangle. An example of the stimulus display is shown on Fig 4. At the two terminals of the rectangle there were two lines, indicating the target area of the movement. The width of the rectangle and the two target areas was 0.77°. The length of the target areas was 1.1°. The distance between their centers was 5.5°. The orientation of the rectangle was either 26° or 170°. The ERP-related stimuli were identical in the two experiments. The task was to move the disk back and forth between the two target areas within the rectangle. We emphasized the fast and accurate movement (i.e., remaining within the rectangle) while alternatingly reaching the target areas. As in Experiment 1, the number of correct moves increased only if both target areas were entered one after the other. The feedback indicated how many times the participant managed to move the disc between the target areas as in Experiment 1, and also included the number of errors when the disc left the rectangle.

#### Measurement of electrical brain activity

The measurement and analysis of the electrical brain activity were identical to those of Experiment 1.

### Results

#### Behavioral performance

Table 3 shows the number of movements in the first and second blocks of the sessions for movements corresponding to the standard (Same) and deviant (Opposite) orientations of the texture elements.

**Table 3:** Behavioral performance in Experiment 2.

	Same	Opposite	Control
first	398.1 (18.0)	397 6 (20.0)	405.8 (14.3)
second	396.5 (19.4)	396.6 (14.4)	399.5 (14.4)

Mean number of movements in the Same, Opposite and Control conditions, in the first and second blocks (*S*.*E*.*M*. in parenthesis).

Performance was measured by the number of goal-directed movements within a single block. In order to assess the possibility of practice, we calculated performance for the first and second blocks of each condition. We calculated a two-way ANOVA with factors of Condition (Same, Opposite, Control), and Blocks (First, Second), where ‘Same’ is the movement direction corresponding to the standard stimuli, and ‘Opposite’ corresponds to the direction of deviant. Neither the main effect of Condition or Block order, nor the interaction were significant. Participants completed 1.67 correct moves/second on average during the whole session.

#### Event-related potentials

**Fig 5:**
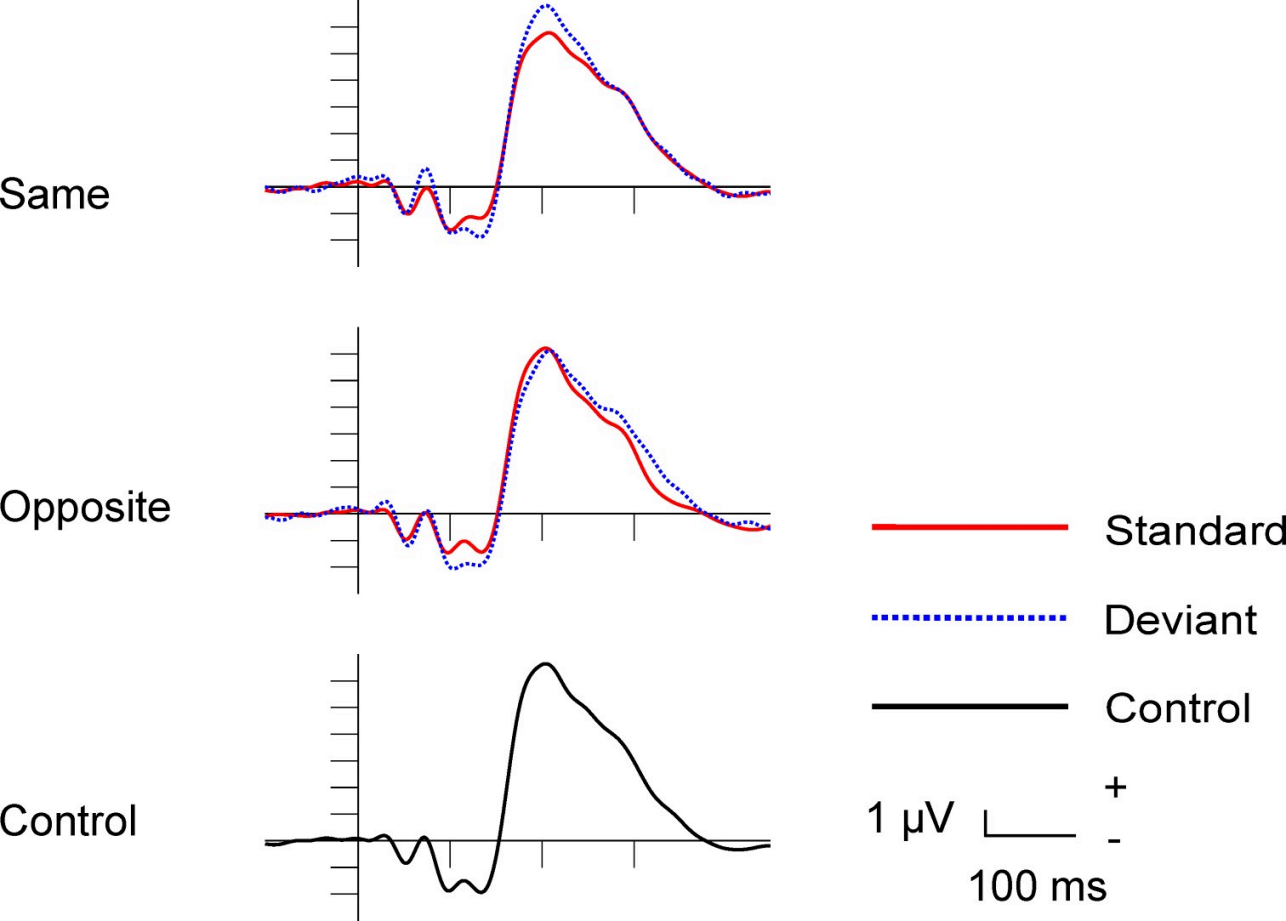
Standard, deviant and control grand-average ERPs in the Same, Opposite and Control conditions in Experiment 2.

The average rejection rate of epochs (eye-blink, eye movement) was 7.68%. Fig 5 shows the standard, deviant and control ERPs in the occipital ROI in the Same and Opposite conditions. Following a very early positivity (P1/C1), we obtained again double negativity (C2/N1) and a positive peak (P2).

#### Difference potentials

**Fig 6:**
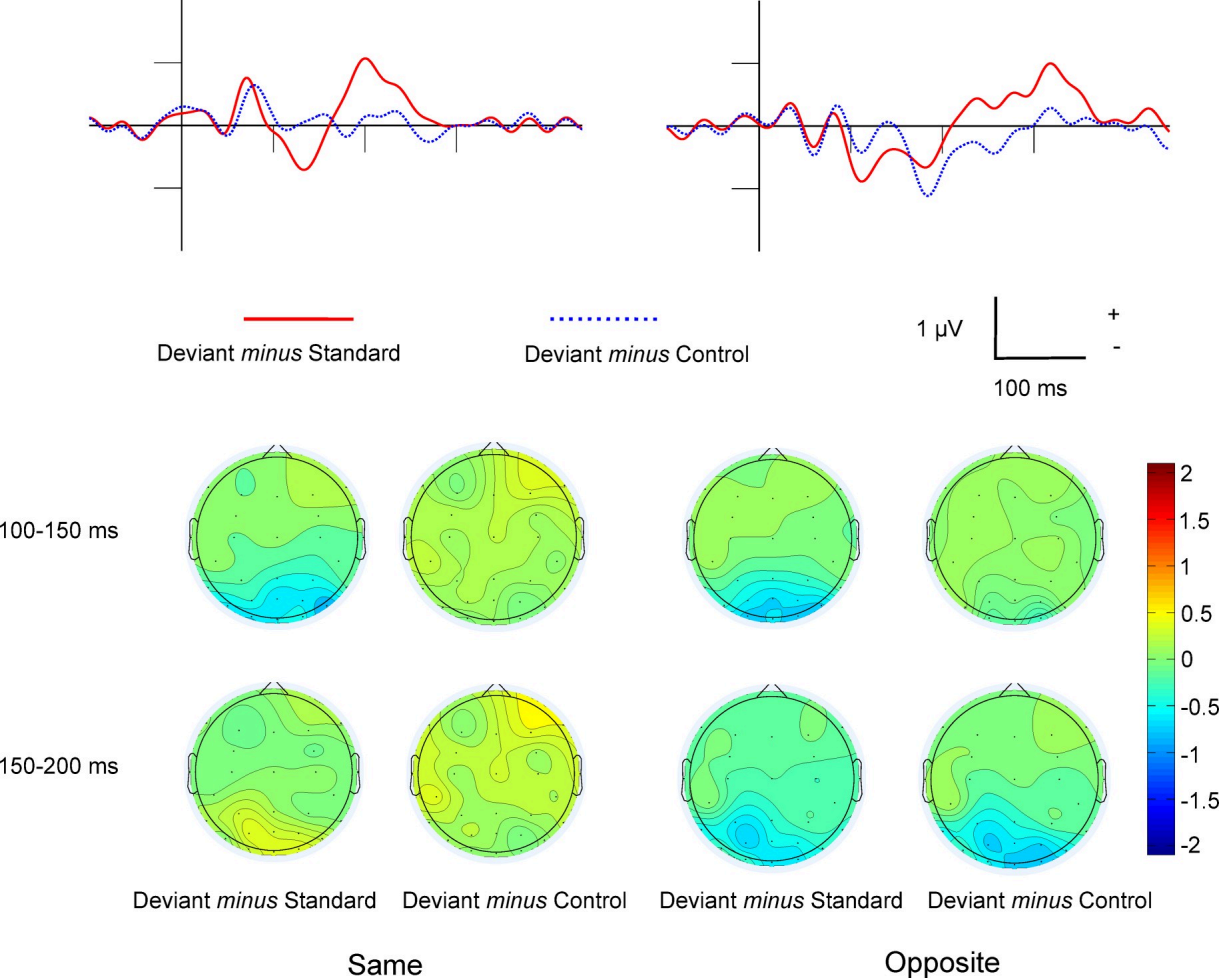
Experiment 2: Difference potentials and surface distributions. The Deviant *minus* Standard and Deviant *minus* Control difference potentials in the occipital ROI and the surface distributions in the Same and Opposite conditions.

Fig 6 shows the Deviant *minus* Standard and Deviant *minus* Control difference potentials in the occipital ROI and the surface distributions. Table 4 shows the mean amplitude values of the 100–150, 150–200 and 200–350 ms ranges, and the significance levels of one-sample t-tests in comparison to zero.

**Table 4:** Amplitude values of the difference potentials in Experiment 2.

	Deviant *minus* Standard		Deviant *minus* Control	
	Same	Opposite	Same	Opposite
100–150 ms	-0.47 (0.13)**	-0.62 (0.20)**	0.03 (0.16)	-0.16 (0.22)
150–200 ms	0.40 (0.18)*	-0.51 (0.19)*	0.01 (0.19)	-0.70 (0.20)**
200–350 ms	0.29 (0.17)	0.48 (0.14)**	0.01 (0.21)	-0.14 (0.17)

Mean amplitude values (μV) of the difference potentials in the 100–150, 150–200 and 200–350 ms range (*S*.*E*.*M*. in parenthesis).

*p<0.05 in t-tests, in comparison to zero.

**p<0.01 in t-tests, in comparison to zero.

As for the Deviant *minus* Standard difference, a negative difference potential emerged within the 100–200 ms range, followed by a long-lasting positivity. As for the Deviant *minus* Control difference potentials, the negativity was restricted to the 150–200 ms range.

In the 100–150 ms range in the two-way ANOVA (Difference x Condition) we obtained a significant Difference main effect, F(1,25) = 15.605, ƞ_p_^2^ = 0.38, p<0.001. The Deviant *minus* Standard difference potential amplitude was -0.54 μV, whereas the Deviant *minus* Control difference amplitude was -0.06 μV.

In the 150–200 ms range the Difference main effect was significant, F(1,25) = 6.246, ƞ_p_^2^ = 0.20, p = 0.019 (the Deviant *minus* Standard difference potential amplitude was -0.05 μV, and the Deviant *minus* Control difference potential amplitude was -0.35 μV, i.e., the latter difference potential was more negative). Besides, the Condition main effect was also significant, F(1,25) = 10,385, ƞ_p_^2^ = 0.29, p = 0.004. In the Same condition the difference potential amplitude was 0.21 μV, whereas in the Opposite condition it was -0.61 μV.

In the 200–350 ms range the main effect of difference was significant, F(1,25) = 26.751, ƞ_p_^2^ = 0.52, p<0.001, the Deviant *minus* Standard difference potential amplitude was 0.38 μV, and -0.07 μV was the Deviant *minus* Control difference potential amplitude. Furthermore, we obtained significant interaction, F(1,25) = 7.381, ƞ_p_^2^ = 0.23, p = 0.012. According to the post-hoc Tukey HSD tests the difference between the Deviant *minus* Standard and Deviant *minus* Control was larger in the Opposite condition. While the Deviant *minus* Standard difference potential amplitude was larger in the Opposite condition, the Deviant *minus* Control difference potential amplitude was larger in the Same condition. However, both differences were less than 0.2 μV, therefore we refrain from discussing this interaction.

### Discussion

In Experiment 2 the negativity in the 100–150 ms range was larger in the Deviant *minus* Standard than in the Deviant *minus* Control difference, i.e., we replicated the results of Experiment 1. Concerning the 150–200 ms range, unlike in Experiment 1, in Experiment 2 the Deviant *minus* Control difference was *more* negative than the Deviant *minus* Standard difference. More importantly, this difference was due to the negativity in the Opposite condition. Accordingly, in this latency range the continuous presence of an oblique rectangle influenced the emergence of ‘genuine’ vMMN. Note, that in the Opposite condition the orientation of the rectangle was different from the orientation of the elements of the frequent texture, and identical to the orientation of the elements of the deviant texture. Thus, the orientation of the rectangle *facilitated* the sensitivity to the rare texture orientation. One might have expected a more efficient mismatch process in the Same condition due to a reinforced representation of the orientation of the standard texture. This assumption was plausible because of a possible increased activation of orientation-specific neural structures elicited by the identical orientation of the rectangle and the texture. However, according to our results the system underlying the adaptation/mismatch processes separated the object (rectangle) from the background. Later (in the 200–350 ms epoch) the positivity of the Deviant *minus* Standard difference was eliminated by the Deviant *minus* Control difference potential, similarly as in Experiment 1.

## General discussion

We measured event-related potentials to task-irrelevant textures of oblique bars. Participants moved the mouse in the direction of the frequent (standard) orientation of bars (Same condition), or in the opposite orientation (Opposite condition). In Experiment 1 the movement direction was cued by two target circles, whereas in Experiment 2 the movement direction was cued by a continuously presented rectangle. Besides standard-deviant (Oddball) blocks we presented equal probability control blocks, i.e., blocks of variable bar-orientation with the same probability of each orientation as the probability of the oddball deviant. Although in Experiment 1 we obtained the expected negative difference potential (Deviant *minus* Standard difference, a posterior negativity in the 100–150 ms range), the movement direction had no effect on it, and this difference was eliminated by the Deviant *minus* Control comparison. In Experiment 2 the 100–150 ms negativity was similar in the Same and Opposite blocks, however in the 150–200 ms range the negativity (preserved in the Deviant *minus* Control difference potential) was confined to the Opposite condition.

Contrary to the results of studies investigating common goal of action and perception, we did not find movement-perception interaction. A typical example of such a connection is the effect of self-generated movement on sensory ERP activity [11–15]. Theoretical accounts of the movement-related ERP effects frequently concentrate on the anticipated perceptual consequence (‘forward model’) [31]. However, as some results show, the ERP effect, usually a diminished auditory N1, is due to a set of movement-contingent and attentional effects [13,32]. The present results indicate that without the contingency of a voluntary action and a visual stimulus, specific low-level visual representations are not activated, at least at the level detected by ERP methods. We suggest that common coding of action and perception involves higher mechanisms of visual processing. These mechanisms may require attentional processing.

The absence of ‘genuine’ vMMN (i.e., the lack of Deviant *minus* Control difference) can be attributed to the adaptation of ERP components of the frequently presented standard, compared to the less adapted, rarely presented deviant (see e.g., [22] in the auditory modality, [20] and [19] in the visual modality). According to an MMN tradition, the Deviant *minus* Standard difference eliminated by the control procedure is due to a refractory process, that is a decreased responsiveness of input structures after prolonged stimulation (for a review see [33], for a discussion see [27] and [34]). The original refractory account did not attribute any functional significance to such amplitude decrements. On the contrary, in other fields such as fMRI research, activity decrease is considered as a functionally significant process, e.g., the consequence of the acquired prediction [35]. Recently auditory and visual MMN is discussed in the predictive coding framework (e.g., [7,36]). According to this framework perceptual systems are prepared for the appearance of high probability events. Processing of such an event does not require the activity of a broad neural network, sparing the energy consumption of the brain [37]. Decreased activity to the standard is a signature of such processes. In this view, mismatch responses (‘genuine’ MMN) of various modalities are error signals, elicited by events requiring further processes.

A further issue is the relation of the difference potentials to the ERP componentry. In the present study Deviant *minus* Standard difference emerged in the negative components (the 100–200 ms range) and also in the P2 range. Over the posterior locations negative deflections are the aggregates of various sources, like pattern-specific components [38] and the N1 subcomponents [39]. The present result indicates that repeated presentation elicits a widespread activity decrease. Maintaining the view that the ERP amplitude decrease is a signature of the acquisition of memory representation, a wide network of posterior structures with feed forward and feedback connections (e.g., [40,41]) is involved in this process.

At the outset we expected that in Experiment 2 the identical orientation of the continuously present rectangle and the frequent orientation of the task-irrelevant textures emphasized the deviant-standard contrast, and if ‘genuine’ vMMN emerged, it would be present in the Same condition. This suggestion was based on low-level processes, i.e., the activity change of elementary orientation-specific structures. On the contrary, ‘genuine’ vMMN emerged only in Experiment 2 in the 150–200 ms range, and only in the Opposite condition. In this condition the orientation of the continuously present object was different from the orientation of the frequent elements, and identical to the orientation of the deviant texture elements.

In some vMMN studies with orientation deviancy, ‘genuine’ vMMN appeared [18,20,42,43], whereas in the File et al. [19] study, and in the majority of ranges and conditions of the present study the equal probability control eliminated the deviance-related (i.e., the ‘genuine’) vMMN effect. In the Astikainen et al. [18] study participants attended to auditory stimuli, while the vMMN-related stimuli appeared at the center of the screen. Even if the vMMN-related stimuli were task-irrelevant, a sole visual stimulus at the center of the screen might capture attention [44]. In the Kimura et al. [20] study the orientation of the bars was unrelated to the task, but participants had to attend to the terminals of these bars. Such task-irrelevant features of task-relevant objects are also processed at a higher level (e.g., [45]). Attentional control was more stringent in the Kimura and Takeda [43] study; in the center of oblique bar patterns participants responded to the occasional size change of the target. However, in this method the target probability is low. Therefore, between two target stimuli participants usually have time for attending to the vMMN-related stimuli. On the contrary, in the File et al. [19] and in the present study the tasks demanded continuous attention to the central task field. We propose that in case of such continuous central fixation, less salient repeated stimuli acquire memory representation (the activity to the oddball standard decreases), but the appearance of a deviant does not initiate a mismatch process (deviants do not elicit larger activity than the equal probability control stimuli). In terms of the predictive memory framework, increasing the saliency of either the standard or the deviant, deviant stimuli initiate a cascade of processes. In this regard the results of Kojouharova et al. [42] are relevant. In this study oblique bars appeared as deviant within the sequence of complex shapes and *vice versa*, i.e., deviants were highly different from the standard, and the bars elicited ‘genuine’ vMMN. In the present study the contrast between the orientation of the continuous rectangle and the texture elements might have played such decisive role in increasing the saliency of vMMN-related stimuli to a level that initiates mismatch processes.

As a limitation, the position of the screen was vertical, whereas the mouse movement was horizontal. However, this is a common arrangement for computer usage, and the equivalence of mouse movement and the visual movement on the screen is a part of everyday practice.

## Conclusions

Frequent orientation of elements of visual texture are automatically registered in visual memory. The continuous presence of an oblique object influences the saliency of contrast between the orientations of elements of the non-attended texture, leading to automatic detection of deviant orientation within the sequence of frequent (standard) orientation of texture elements. We obtained no evidence that the congruence or incongruence of direction of voluntary movement influences the detection or the identification of deviant orientation of texture elements.
